# Impathy and Emotion Recognition: How Attachment Shapes Self- and Other-Focused Emotion Processing

**DOI:** 10.3390/brainsci15050516

**Published:** 2025-05-18

**Authors:** Dirk W. Eilert, Karin de Punder, Jeff Maerz, Johanna Dose, Manuela Gander, Philipp Mensah, Stefanie Neubrand, Josef Hinterhölzl, Anna Buchheim

**Affiliations:** 1 Institute of Psychology, University of Innsbruck, 6020 Innsbruck, Austria; karin.de-punder@uibk.ac.at (K.d.P.); jeff.maerz@uibk.ac.at (J.M.); manuela.gander@uibk.ac.at (M.G.); anna.buchheim@uibk.ac.at (A.B.); 2 Eilert-Academy, 13585 Berlin, Germany; philipp.mensah@eilert-akademie.de; 3 University Clinic for Psychiatry II, Department of Psychiatry, Psychotherapy, Psychosomatics and Medical Psychology, Medical University of Innsbruck, 6020 Innsbruck, Austria; johanna.dose@tirol-kliniken.at (J.D.); josef.hinterhoelzl@tirol-kliniken.at (J.H.); 4 University Clinic for Child and Adolescent Psychiatry, Medical University of Innsbruck, 6020 Innsbruck, Austria; 5 School of Health, Education and Social Sciences, SRH University of Applied Sciences Heidelberg, 69123 Heidelberg, Germany; stefanie.neubrand@srh.de

**Keywords:** attachment representation, impathy, emotion recognition, defensive exclusion, personality disorders, adult attachment projective picture system, emotion processing

## Abstract

**Background/Objectives:** Early attachment experiences and psychopathology both shape individuals’ emotion processing. However, the specific influence of adult attachment representations on self- (intrapersonal) and other-focused (interpersonal) emotion processing remains unclear, particularly in the context of personality disorders. This study examined how attachment representations (organized vs. unresolved) modulate intrapersonal emotion perception (“impathy”) and interpersonal emotion recognition while accounting for personality pathology. **Methods:** Thirty-three adults (twenty-four patients with a personality disorder and nine healthy controls) were assessed for attachment representation using the Adult Attachment Projective Picture System (AAP). Emotion processing was measured via the Impathy Inventory and a facial emotion recognition task (READ-64). Group differences (organized vs. unresolved attachment; patients vs. controls) and correlations with the severity of unresolved attachment status were analyzed. **Results:** Patients with organized attachment representations did not differ from healthy controls in emotion recognition but showed significantly reduced impathy (*M* difference = −21.72, *SE* = 6.20, *p* = 0.002, 95% CI [−34.42, −9.01], *d* = −1.57). In contrast, patients with unresolved attachment exhibited impairments in both intrapersonal (*M* difference = −32.99, *SE* = 6.20, *p* < 0.001, 95% CI [−45.69, −20.29], *d* = −2.39) and interpersonal (*M* difference = −12.37, *SE* = 4.36, *p* = 0.008, 95% CI [−21.28, −3.46], *d* = −1.23) emotion processing compared to healthy controls. Furthermore, the severity of unresolved attachment status correlated with greater impairment in recognizing anger (*r* = −0.74, *p* = 0.004). **Conclusions:** An organized attachment representation may act as a protective factor, preserving interpersonal emotion recognition even in the presence of psychopathology. Conversely, an unresolved attachment constitutes an additional risk factor that exacerbates emotion processing impairments in the context of personality pathology. Attachment representation thus emerges as an active modulator of core emotion processes, with important implications for theory and targeted interventions in personality disorders.

## 1. Introduction

Attachment theory claims that attachment experiences in early childhood play a fundamental role in the emotional development of the self- and, therefore, in the development of the individual nature of emotion processing [1,2,3]. It was John Bowlby, the “father of attachment theory”, who emphasized the importance of emotions in the development of the so-called *internal working model of attachment* ([4], p. 157): “During the earliest years of our lives, indeed, emotional expression and its reception are the only means of communication we have, so that the foundations of our working models of self and attachment figure are perforce laid using information from that source alone”. A systematic review [5] confirmed this notion: across all objective measures of emotion (autonomic nervous system, brain activity, biochemical level, or nonverbal behavior), there is a significant connection between the patterns of attachment representation in adults and the quality of emotion regulation in the face of stress, and the spontaneous facial expression of emotion. Therefore, the individual attachment representation is one promising factor that can deepen our understanding of the adaptive processing of emotions and, thus, a promising target for interventions fostering it. However, emotion regulation and the spontaneous facial expression of emotion are just two aspects of emotion processing. Emotion processing also comprises the intrapersonal and interpersonal perception of emotion.

Since impairments in emotion processing (including emotion perception) are a common denominator for high comorbidity of mental disorders [6] and a potential underpinning of personality pathology [7], and further, the adaptiveness of emotion processing is crucial for social functioning in healthy [8] and clinical populations [9], the present study aims to deepen the understanding of attachment as a central target point for fostering the functional processing of emotions in all its aspects, by examining the link between attachment patterns and the intrapersonal and interpersonal perception of emotions.

### 1.1. Self-Focused Emotion Processing: Intrapersonal Emotion Perception

Intrapersonal emotion perception describes the ability to share in and understand one’s own emotions. According to Neubrand and Gaab [10], this ability, which Neubrand [11] calls introversive empathy or *impathy* for short, comprises four dimensions: (1) Perceiving: directing one’s attention inward to take part in one’s own thoughts, feelings, and bodily sensations. (2) meta-position: The ability to perceive one’s feelings but not to merge with them, i.e., not to over-identify with them. (3) accepting attitude: being able to approach one’s own inner phenomena with openness and acceptance. (4) understanding: intentionally engaging with one’s own psychological and physiological phenomena and thereby increasing the degree of insight into these phenomena.

Research confirms the link between individual attachment patterns and the ability to impathize, i.e., to perceive one’s own emotions in relation to the four dimensions as defined above. For the two dimensions of *perceiving* and an *accepting attitude*, studies show a clear association between insecure attachment and an ability impairment in the perception of one’s emotions (e.g., [12,13,14]) and an accepting attitude to one’s inner experience (e.g., [15,16,17]). The construct of reflective functioning according to Fonagy et al. [18] is conceptualized as the ability to understand and interpret one’s behavior and that of others through mental states, also known as mentalization (for an overview of current research, see [19]). This includes the understanding of mental processes (including emotions) and simultaneously emphasizes the importance of the meta-position for successful mentalization. Studies demonstrated a positive link between secure attachment and reflective functioning [19]. In addition, low reflective functioning correlated with a variety of psychopathological diagnoses [20]. Thus, research indicates that attachment is a key factor influencing intrapersonal emotion perception; however, to date, no study has specifically addressed the two impathy dimensions, *meta-position*, and *understanding*, in the context of adult attachment patterns.

### 1.2. Other-Focused Emotion Processing: Interpersonal Emotion Perception

Interpersonal emotion perception refers to the ability to recognize and correctly interpret other people’s emotions. Different forms of empathy need to be distinguished here. Neuroscientific studies showed that cognitive empathy (“I see and understand what you feel”) activates different neural networks than affective empathy (“I feel what you feel”), so two distinguishable abilities can be assumed [21]. Cognitive empathy comprises the Theory of Mind (ToM), perspective-taking, and emotion recognition. Affective empathy, on the other hand, refers to experiencing the emotions of others, also known as emotional contagion [22]. The two types of empathy not only differ on the behavioral level and neuronal activation patterns but also in their correlation with the ability to regulate emotions. While higher cognitive empathy is associated with improved emotion regulation, greater affective empathy is linked with increased difficulties in emotion regulation [23]. Therefore, subsequently, we focus on cognitive empathy and its link to attachment.

Research has demonstrated that attachment patterns influence interpersonal emotion perception. For example, Steele et al. [24] showed that a secure attachment quality at the age of one year (measured using the Strange Situation Procedure, according to Ainsworth [25]) predicts better emotion recognition in infants at the age of six years. Attachment research also found that disorganized attachment in children correlates with impaired emotion recognition ability [26]. In addition, Gallistl et al. [27] showed that AAI-based attachment security (versus insecurity) in adults relates to an enhanced ability to exhibit cognitive empathy. Another study using self-report measures found significant differential associations between attachment styles and interpersonal emotion regulation [28].

Although previous research indicates attachment is important for interpersonal emotion regulation processes and perception, there is a lack of research examining the association between adult attachment representation measured by narrative interviews capturing unconscious processes (versus self-report questionnaires) and interpersonal emotion perception. Meta-analyses (e.g., [18]) have shown that there is only a trivial to small overlap between conscious self-report attachment questionnaires and narrative attachment interviews, which are designed to “surprise” the unconscious. The present study with healthy individuals and patients with a personality disorder fills this gap by measuring the individual attachment representation using the Adult Attachment Projective Picture System (AAP), an instrument based on analyzing narratives elicited in response to theoretically derived attachment-relevant drawings.

### 1.3. Defensive Exclusion as a Possible Mechanism for Impairing Emotion Perception

Beyond examining the correlations of individual attachment representation with intra- and interpersonal emotion perception, the question also arises as to what possible attachment-related mechanisms might impair emotion perception as a result of disorganized/unresolved attachment.

According to attachment theory, attachment disorganization develops through behavioral tendencies experienced as incompatible. If the attachment figure is experienced as a needed haven of safety and, simultaneously, as a source of threat, the infant experiences a state described by Main and Hesse [29] as “fright without solution”, caused by perceiving its attachment figure as either frightening (e.g., through violent behavior that is often accompanied by an expression of anger) or frightened (e.g., through an expression of fear resulting of a traumatized state). In accordance with findings presented by Bowlby [3], in such a situation, defense processes are activated to resolve the inner conflict of “fright without solution” or at least make it bearable. Through *defensive exclusion*, certain information is excluded from consciousness; specifically, information incompatible with existing mental models [30], such as the facial expressions of *anger* or *fear* that cause the infant to experience a threat [30]. To maintain the experience of the attachment figure as a source of security instead of a threat, the anger or fear perceived in the context of the attachment experience is considered to be dissociated. This is in line with findings of attachment research in adults, demonstrating that mothers with unresolved attachment representation (the adult equivalent to childhood disorganized attachment) react to emotional stress of their infants with a “blunted” amygdala response [31]. On the mother’s side, this indicates a diminished empathic response to her infant’s stress; thus, a defensive response.

In the Adult Attachment Projective Picture System (AAP), the defense processes introduced and described by Bowlby [3] are operationalized by George and West [32] to derive the attachment classification from the coding at the level of the words in the attachment narrative (i.e., the story that the person tells about the AAP pictures). In the AAP, three defense processes are distinguished: (1) *deactivation*, which is connected to insecure–dismissing attachment representation; (2) *cognitive disconnection*, which is linked to insecure–preoccupied attachment representation; and (3) *segregated systems*, which, if not resolved by attachment resources, are associated with unresolved attachment, i.e., “disorganized” attachment in adults. Since the defense process of segregated systems is conceptualized as the product of defensive exclusion in which trauma-related memories and emotions are encoded in a separate representational model that is inaccessible to consciousness ([32], p. 89), its impact on the impairment of emotion perception should be the strongest among the above-defined defense processes. Therefore, the present study focuses on examining defensive exclusion through segregated systems in adults.

Studies that examined the segregated systems defense process have demonstrated that the severity of an unresolved attachment status is associated with increased personality pathology [33,34] and increased non-suicidal self-injurious behavior [35], a common feature of Borderline Personality Disorder. Based on these findings, the present study investigates this approach in the context of emotion perception in patients with personality disorders. For the field of interpersonal emotion perception, we hypothesize that the more severe a person’s unresolved attachment status, the more impaired the recognition should be in relation to emotions that signal potential threats to their sense of self or interpersonal relationships. Research shows that facial expressions of fear and anger play a central role in perceiving such social threats [36].

So far, there are no studies that have examined the correlation of attachment representations with interpersonal emotion perception in consideration of attachment-related defense processes. A deeper understanding of this link can provide valuable insights for psychotherapy in general and for emotion-focused interventions in particular. For example, their effectiveness can be increased by tailoring such interventions focusing on the individual “segregated affective systems” (e.g., anger and/or fear).

### 1.4. Aim of the Present Study and Hypotheses

The present study examines the connection between individual attachment representation and the nature of a person’s intra- and interpersonal emotion perception, while accounting for personality pathology. A deeper understanding of factors influencing emotion processing may provide valuable insights for psychotherapy in general and for emotion-focused interventions in particular.

Regarding the area of **intrapersonal emotion perception**, the following hypothesis is formulated based on the above-described research results:

**H1.**  *“Individuals with unresolved attachment representation exhibit poorer impathy than individuals with organized attachment representation (with or without personality disorder)”.*

According to the above-mentioned literature results, the following hypotheses were formulated in the area of **interpersonal emotion perception**:

**H2.**  *“Individuals with unresolved attachment representation exhibit poorer emotion recognition ability than individuals with organized attachment representation (with or without personality disorder), specifically for fear and anger”.*

**H3.**  *“The more severe the unresolved attachment status, the more impaired the facial emotion recognition of fear and anger”.*

This study extends previous research by integrating a narrative-based assessment of adult attachment representation (Adult Attachment Projective Picture System, AAP) with both self- and other-focused emotion processing in a clinical population diagnosed with personality disorders. While earlier studies often relied on self-report measures of attachment, which primarily assess conscious evaluations of the quality of their past and present relationships, the AAP is designed to elicit and analyze unconscious defensive processes through narrative responses to theoretically derived attachment-relevant pictures. This enables a more nuanced and externally coded understanding of internal working models of attachment. Moreover, the inclusion of the concept of impathy as a multidimensional operationalization of intrapersonal emotion perception introduces a novel construct to the field of attachment and personality research.

## 2. Materials and Methods

### 2.1. Participants

The present sample is a subsample from a larger ongoing study at the University of Innsbruck (Innsbruck, Austria) and the Medical University of Innsbruck (Innsbruck, Austria), which started in May 2024. The sample comprises 33 participants aged between 18 and 25 (*M* = 21.52, *SD* = 2.12), primarily female (69.7%, *n* = 23), and includes both patients (*n* = 24) and a control group (*n* = 9) consisting of psychology students from the University of Innsbruck. The clinical sample was recruited from a day-treatment program for young adults, and inclusion criteria required participants to be between 18 and 25 years of age. To ensure comparability, healthy controls were selected within the same age range.

Patients diagnosed with a personality disorder according to DSM-5 (AMPD) and the model of personality organization [37], and with sufficient proficiency in German, were recruited through the Department of Psychiatry, Psychotherapy, Psychosomatics and Medical Psychology at the Medical University of Innsbruck. Exclusion criteria included acute psychosis, severe substance abuse, organic mental disorders, intellectual disability, severe physical illness, and pregnancy. Table 1 gives an overview of the sociodemographic and clinical characteristics of the present sample.

### 2.2. Procedures

For the present study, the subjects signed an informed consent form and completed the questionnaires. The questionnaires included an acquisition of sociodemographic data, the Symptom-Checklist-90-Standard (SCL-90-S) [38], and the Impathy Inventory created by Neubrand [11]. Then, a trained psychologist conducted an attachment interview using the Adult Attachment Projective Picture System (AAP) according to George and West [39]. After the AAP, the subjects performed the READ-64 Test (Reliable Emotional Action Decoding Test, 64-item version) in accordance with the findings of Eilert [40].

The present study was conducted in accordance with the Declaration of Helsinki and approved for studies involving humans by the Institutional Ethics Committee of the Medical University of Innsbruck, Innsbruck, Austria (approval number 1395/2023).

### 2.3. Measures

#### 2.3.1. Adult Attachment Projective Picture System (AAP)

The Adult Attachment Projective Picture System (AAP) developed by George and West [32] follows the Bowlby and Ainsworth tradition of assessing attachment. As a well-validated instrument, the AAP assesses attachment representations in adolescents and adults. The methodological foundation consists of analyzing narratives elicited in response to theoretically derived attachment-relevant drawings. These illustrations depict scenes of solitude, illness, separation, and death. The stimulus material comprises drawings featuring adults and children in solitary situations (monadic pictures representing abandonment), adult–adult/adult–child dyads (dyadic pictures representing interpersonal distress), and one neutral picture. Participants are instructed to tell a story for each image, guided by standardized interview questions.

The AAP coding system is based on a constructivist–developmental framework and evaluates both the narrative content and defensive processes. Beyond the categorical attachment classification, the coding includes markers for defensive exclusion strategies (deactivation, cognitive disconnection, and segregated systems). Segregated systems, in particular, signal the presence of unresolved trauma-related material such as disorganized themes, helplessness, or disintegration in the narrative. These markers are counted to assess the severity of attachment dysregulation. Coders assess whether the character in the story demonstrates access to internalized attachment resources, such as help-seeking, comfort, or meaning-making, or whether narrative breakdown occurs under conditions of attachment distress. When characters cannot integrate or contain the segregated markers and remain in a threatening or dangerous attachment situation, these markers are identified as unresolved (U characteristics, ≥0). Thus, the AAP provides both a categorical classification and a dimensional indicator of unresolved attachment.

Classification is determined by evaluating response patterns across the entire series of pictures, with each response assessed for content and defensive processes. Organized attachment in the AAP is defined as secure, insecure–dismissing, and insecure–preoccupied classification—consistent with attachment theory analogous to Ainsworth’s four categories for attachment quality in children [25] and the attachment representations in the Adult Attachment Interview (AAI) [41,42]. In the AAP, the term “unresolved” is coded when an individual’s narrative reveals an inability to seek comfort from a caregiver or protect themselves in response to traumatic stressors such as fear, isolation, or death. During the AAP interview, attachment distress remains unprocessed and cannot be effectively contained or reintegrated by the individual. A construct-based coding dimension [43,44] enables the assessment of the severity of attachment trauma by counting the occurrence of unresolved trauma-related material in the subject’s narrative of the AAP interview.

Multiple studies have provided strong evidence for the psychometric robustness of the AAP in both adult [32,44,45] and adolescent populations [46]. Several studies report good interrater reliability, with a concordance rate of 90% (κ = 0.85, *p* < 0.001) [44,46,47]. Additionally, the AAP has demonstrated discriminant validity in controls and clinical patients [32,47] and exhibits strong test–retest reliability over three months (*r* = 0.84). Further support for its validity comes from studies showing a high level of agreement with the Adult Attachment Interview (AAI). Specifically, concordance rates for the four-category attachment classification (secure, insecure–dismissing, insecure–preoccupied, and unresolved) reached 90% (κ = 0.84, *p* < 0.001), while the secure vs. insecure classification yielded a concordance rate of 97% (κ = 0.89, *p* < 0.001) [32,48].

To provide additional context, readers are referred to published examples of AAP illustrations and corresponding narrative excerpts (e.g., Buchheim et al. [49]), which illustrate the narrative differences between organized and unresolved attachment representations. Including visual or narrative examples can offer further insight into the coding rationale and clinical relevance of the AAP.

#### 2.3.2. Symptom-Checklist-90-Standard (SCL-90-S)

The SCL-90-S is a self-report questionnaire designed to assess subjective impairment caused by somatic and psychological symptoms experienced over the past seven days. It consists of 90 items, rated on a 5-point Likert scale ranging from (0) *not at all* to (4) *very strong*. Example items include “Nervousness or shakiness inside”, “Unwanted thoughts that won’t leave your mind”, and “Feeling that most people cannot be trusted”. The questionnaire evaluates psychological distress across nine subscales, including aggressiveness/hostility, anxiety, depressiveness, paranoid thinking, phobic anxiety, psychoticism, somatization, insecurity in social contact, and obsessiveness. In addition to these subscales, three global indices provide an overarching measure of psychological distress: the Global Severity Index (GSI), which reflects overall psychological burden; the Positive Symptom Distress Index (PSDI), which assesses the intensity of reported symptoms; and the Positive Symptom Total (PST), which indicates the number of symptoms perceived as distressing.

In this study, the Global Severity Index (GSI) of the SCL-90-S was used as an indicator of overall psychological distress to contextualize participants’ clinical symptom burden. While it was not included as a covariate in the main analyses to avoid over-adjustment, given the overlap with personality pathology, the GSI provides important background information that will allow us to better understand group differences in relation to attachment representation in controls and patients.

Psychometric evaluations indicate that the SCL-90-S shows satisfactory to excellent reliability [38]. Internal consistency, as measured by Cronbach’s alpha, ranges between 0.76 and 0.97, and test–retest reliability over a one-week period shows satisfactory to good values, supporting its applicability for repeated measurements. The validity is supported by meaningful correlations with other established psychodiagnostic instruments, further confirming its ability to assess psychological distress comprehensively [50].

#### 2.3.3. Impathy Inventory

The Impathy Inventory assesses impathy as a personality trait using a 5-point Likert scale with alternatives ranging from (0) *strongly disagree* and (4) *strongly agree.* The self-report questionnaire was thoroughly developed in a multi-stage development process applying theoretical and data-based approaches. Preliminary study results [11] show a very good internal consistency of the total scale with Cronbach’s α = 0.92. The four subscales demonstrate good internal consistency: Perceiving (α = 0.81), meta-position (α = 0.86), accepting attitude (α = 0.86), and understanding (α = 0.78). The Impathy Inventory comprises 20 items that are evenly distributed across these four subscales. Three sample items (translated from German by the authors) include, “I engage with my feelings”, “I can look at my feelings and thoughts without judging them”, and “If I am going through a very difficult time, I can intentionally turn towards or away from my feelings”. The original questionnaire was validated in German; the English wording is provided for illustrative purposes. Subscale scores and a total impathy score were calculated.

Correlations found with measures of related psychological constructs, i.e., self-esteem, emotional intelligence, and empathy, are all within a range that supports the discriminant validity of the impathy inventory. Furthermore, significant negative correlations with anxiety (*r* = −0.66, *p* < 0.001) and negative affect (*r* = −0.42, *p* < 0.001), as well as positive correlations with positive affect (*r* = 0.56, *p* < 0.001) and life satisfaction (*r* = 0.48, *p* < 0.001), provides initial evidence to support the criterion validity of the scale. These findings suggest that the Impathy Inventory is a psychometrically sound and reliable self-report instrument for assessing introversive empathy.

#### 2.3.4. Reliable Emotional Action Decoding Test, 64-Item Version (READ-64 Test)

The READ-64, an updated version of the READ-49 [40], measures emotion recognition ability (interpersonal emotion perception) through a facial emotion recognition performance test. The 64 items are divided into eight dimensions that refer to seven purely facial emotions, i.e., emotions that, according to research [51,52], can be recognized purely by facial expressions (in contrast to multimodal emotions such as pride): fear, surprise, anger, disgust, contempt, sadness and happiness, as well as social smiles as an additional eighth dimension. According to previous research findings, emotions are less frequently displayed in everyday life in full facial expressions and more often as subtle expressions [53,54]. To account for this, the items for each emotion category are composed of four subtle expressions (in which the emotion is not displayed as a full expression, but only as a partial expression or in a lower intensity facial expression) and four full facial expressions.

The internal consistency of the READ-64 total scale for the present sample can be considered acceptable to good with Cronbach’s α = 0.77. The eight subscales range from Cronbach’s α = 0.56 (anger) to 0.77 (happiness). The internal consistency (including the subscales) is thus within the range of Cronbach’s α = 0.47 (DANVA Voices; Diagnostic Analysis of Nonverbal Accuracy) to 0.83 (GERT-S; Geneva Emotion Recognition Test, short version) of other tests of emotion recognition [55].

The construct validity is supported by the correlation of the READ with the Mayer–Salovey–Caruso Emotional Intelligence Test (MSCEIT), *r* = 0.43 (*p* = 0.002) [56]. The MSCEIT is an ability-based assessment that measures emotional intelligence (for an overview, see [57]). The ability to recognize emotions, which the READ measures, can be understood as a sub-ability of emotional intelligence, which the MSCEIT measures. Moreover, two studies demonstrated that the READ can indicate improvements in emotion recognition ability caused by a 10-day training program in emotion recognition [58,59]. The test–retest reliability was confirmed using two samples [58,59] synthesized for the calculation (*r* = 0.70, *p* < 0.001). The results demonstrate that the READ shows good test–retest reliability and measures both stable interindividual differences in emotion recognition ability and situational fluctuations due to external factors. These findings suggest that the READ-64 is a psychometrically valid and reliable measure of emotion recognition ability.

### 2.4. Statistical Analysis

The data analysis was conducted with IBM SPSS Statistics (Version 30.0.0.0). The significance level was set at *p* < 0.05 for all applied analyses. General linear models were applied to test for group differences in psychopathology, impathy, and emotion recognition ability between organized controls, organized patients, and unresolved patients (Table 2). Levene’s test was used to assess homogeneity of variance. Effect sizes are reported as a partial eta squared (*η*^2^*_p_*) for ANOVA models. As gender and age can influence emotion perception ability [60,61], additional adjusted analyses included these as covariates. Pairwise comparisons were hypothesis-driven and, therefore, not adjusted for multiple testing. This decision follows methodological recommendations suggesting that corrections for multiple comparisons are not always appropriate in confirmatory, hypothesis-based research designs, especially when statistical power is limited [62]. Pearson correlation coefficients (Pearson’s r) were calculated to examine associations between the severity of unresolved attachment status (operationalized via the number of unresolved segregated systems markers in the AAP attachment narrative) and fear and anger recognition performance. All analyses were two-tailed.

## 3. Results

### 3.1. Sample Characteristics

Table 2 shows group differences in impathy, emotion recognition ability, and psychopathology between organized controls, organized patients, and unresolved patients. As expected, psychopathology symptoms were significantly higher in both organized (*p* < 0.001) and unresolved (*p* < 0.001) patients compared to healthy controls. No significant difference in symptom severity was observed between unresolved and organized patients (*p* = 0.12).

### 3.2. Hypothesis Testing

#### 3.2.1. Intrapersonal Emotion Perception: Impathy

**Figure 1:**
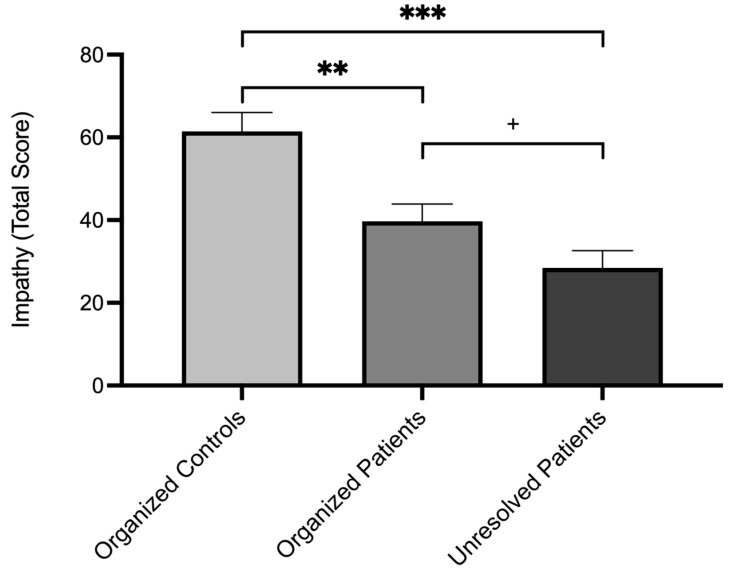
Unadjusted mean scores for impathy (total) in organized controls, organized patients, and unresolved patients. Error bars show standard errors of the mean. *** *p*-value < 0.001, ** *p*-value <0.01, ^+^ *p*-value < 0.10 (marginal).

A significant group difference was observed for the *impathy total score* (*F*_1, 28_ = 14.39, *p* < 0.001, *η*^2^*_p_* = 0.51). Post hoc pairwise comparisons revealed that unresolved patients had a significantly lower score than organized controls (*M* difference = −32.99, *SE* = 6.20, *p* < 0.001, 95% CI [−45.69, −20.29], *d* = −2.39). The difference between unresolved and organized patients was marginally non-significant (*M* difference = −11.27, *SE* = 5.88, *p* = 0.066, 95% CI [−23.33, 0.78], *d* = −0.82). There was also a significant difference in the *impathy total score* between organized patients and organized controls (*M* difference = −21.72, *SE* = 6.20, *p* = 0.002, 95% CI [−34.42, −9.01], *d* = −1.57). For a graphical illustration of the results, see Figure 1. All significant findings remained significant after controlling for gender and age.

**Figure A1:**
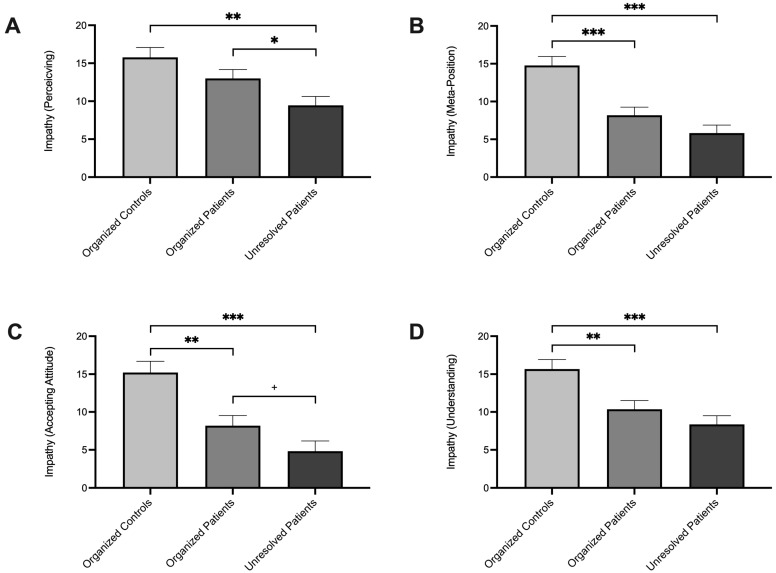
Unadjusted mean scores for all four dimensions of impathy across groups: (**A**) perceiving, (**B**) meta-position, (**C**) accepting attitude, and (**D**) understanding. Error bars show standard errors of the mean. *** *p*-value < 0.001, ** *p*-value < 0.01, * *p*-value < 0.05, ^+^ *p*-value < 0.10 (marginal).

Exploratory analyses indicated significant group effects for all impathy dimensions, with organized and unresolved patients exhibiting significantly lower scores than healthy controls (see Table 2 and Figure A1 in the Appendix A). In all dimensions except for *perceiving*, here, unresolved patients had significantly lower scores compared to organized controls (*M* difference = −6.32, *SE* = 1.74, *p* = 0.001, 95% CI [−9.88, −2.77], *d* = −1.64) and organized patients (*M* difference = −3.55, *SE* = 1.65, *p* = 0.04, 95% CI [−6.92, −0.17], *d* = −0.92). No significant difference was found between organized controls and organized patients (*p* = 0.12).

#### 3.2.2. Interpersonal Emotion Perception: Emotion Recognition

Univariate analyses indicated significant group effects on emotion recognition (*F*_1_, _30_ = 4.63, *p* = 0.02, *η*^2^*_p_* = 0.24). Post hoc pairwise comparisons showed that unresolved patients scored lower in emotion recognition (*READ total score*) than both organized controls (*M* difference = −12.37, *SE* = 4.36, *p* = 0.008, 95% CI [−21.28, −3.46], *d* = −1.23) and organized patients (*M* difference = −9.18, *SE* = 4.12, *p* = 0.03, 95% CI [−17.60, −0.76], *d* = −0.91). Controlling for gender and age did not affect the significance level of these findings.

**Figure 2:**
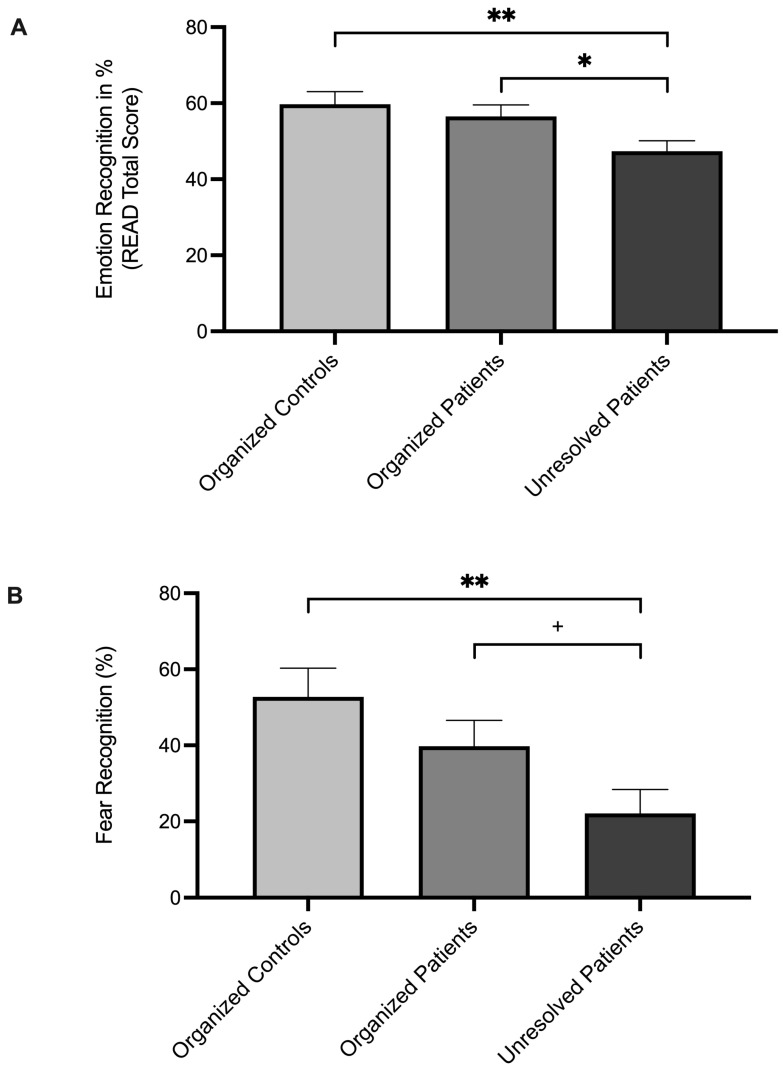
Unadjusted mean emotion recognition (% correct) for (**A**) overall emotion recognition and (**B**) fear recognition in organized controls, organized patients, and unresolved patients. Error bars show standard errors of the mean. ** *p*-value < 0.01, * *p*-value < 0.05, ^+^ *p*-value < 0.10 (marginal).

No significant difference was found between organized controls and organized patients (*p* = 0.49) (see also Table 2 and Figure 2A).

When further exploring the READ-64 dimensions, group differences were observed in *fear recognition* (*F*_1, 30_ = 5.11, *p* = 0.01, *η*^2^*_p_* = 0.25). Pairwise comparisons revealed a significantly weaker performance in fear recognition of unresolved patients compared to organized controls (*M* difference = −30.66, *SE* = 9.77, *p* = 0.004, 95% CI [−50.62, −10.71], *d* = −1.36). In addition, the difference between unresolved and organized patients was marginally non-significant (*M* difference = −17.66, *SE* = 9.23, *p* = 0.065, 95% CI [−36.51, 1.20], *d* = −0.78). No significant differences were found between organized controls and organized patients (*p* = 0.21) (see Table 2 and Figure 2B).

#### 3.2.3. Severity of Unresolved Attachment Status and Fear and Anger Recognition

**Figure 3:**
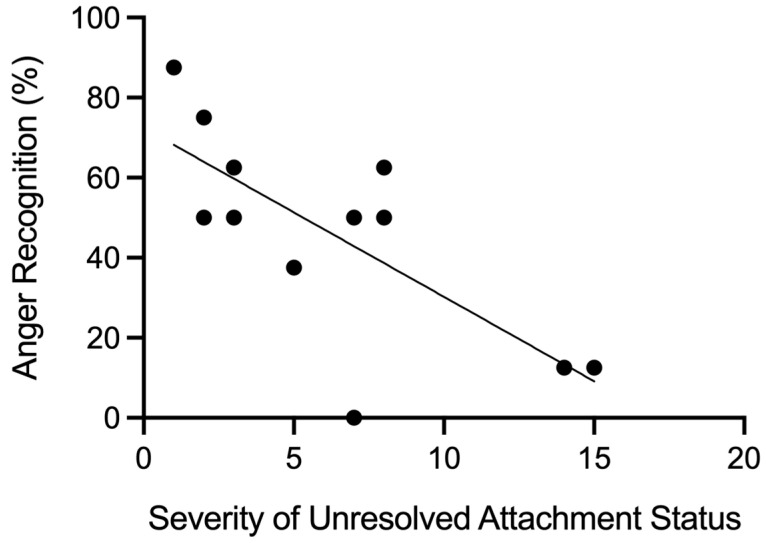
Scatterplot showing the negative correlation between the severity of unresolved attachment status and anger recognition (%) in unresolved patients (*r* = −0.74, *p* = 0.004). Higher trauma scores were associated with lower performance in emotion recognition of anger.

In the group of unresolved patients, a significant negative correlation was found between the number of unresolved segregated systems markers in the AAP attachment narrative (corresponding to the severity of unresolved attachment status) and *anger recognition*, *r* = −0.74, *p* = 0.004 (see Figure 3), but not fear recognition (*r* = 0.36, *p* = 0.22). Also, no significant correlations were observed between the number of unresolved segregated markers and the other dimensions of the READ-64 (all *p*-values > 0.05).

## 4. Discussion

### 4.1. Interpretation of Results

The ability to perceive and process one’s own emotions and those of others is a key foundation for mental health and building and maintaining fulfilling relationships. Numerous studies suggest that both mental disorders and early attachment experiences shape these emotional competencies. In the present study, we investigated the influence of attachment representation on intrapersonal and interpersonal emotion processing, taking clinical symptoms of personality disorders into account.

The results demonstrate that in patients with a personality disorder, an organized attachment representation was associated with an impairment of the perception of one’s own emotions (impathy), while an unresolved attachment was, as we hypothesized, related to impairments in both self-perception and the perception of emotions in others—for the latter, particularly with regard to affective-social danger stimuli such as fear and anger. These findings illustrate the central importance of understanding attachment representation not only as a relationship-relevant factor but also as an active modulator of core emotion processes (as Bowlby, the “father of attachment theory”, already described [4])—with far-reaching implications for theory, diagnostics, and psychotherapy.

To disentangle the specific influence of attachment representation and psychopathology on emotion processing, the sample was split into three distinct groups for the analysis, differing in both clinical status and attachment representation.

The *comparison of organized healthy subjects (controls) with organized patients* primarily demonstrates the **influence of psychopathology**—in this case, personality disorder. The difference in psychopathological load was evident in the SCL-90 scores (Global Severity Index; see Table 2), with severe psychopathological symptoms observed among patients. The comparison between these two groups revealed differentiated results regarding emotion perception: In terms of emotion recognition performance, organized patients did not differ from the healthy subjects. Their ability to recognize and accurately interpret emotions in other people’s facial expressions remained intact. However, differences were demonstrated in their capacity for impathy (introversive empathy). Their entire impathy was impaired significantly compared to healthy subjects. This specifically affected three impathy dimensions: meta-position, accepting attitude, and understanding, all with strongly decreased scores (see Table 2 and Figure A1).

This suggests that the patient’s personality disorder is primarily associated with impaired perception and processing of their own emotions, while interpersonal emotion recognition may be largely preserved in patients with organized attachment representation [63]. Based on these results, we might conclude that an organized attachment representation offers a certain protective function for interpersonal emotion processing despite mental illness.

The *comparison of organized versus unresolved attachment patients* isolated the **influence that attachment representation has on emotion perception beyond psychopathology**. Here, the results showed that patients with unresolved attachment representations were the most severely impaired in terms of both emotion recognition and impathy compared to the other two organized attachment groups. As hypothesized, this suggests the impact of an unresolved attachment, especially on interpersonal emotion perception, but on intrapersonal as well. For the general emotion recognition performance, the link to unresolved attachment was distinct because there was no difference between the organized attachment groups, but between organized and unresolved patients. Therefore, we might consider that there is a link between unresolved attachment representation and interpersonal emotion perception rather than with psychopathology.

Regarding the ability to impathize, unresolved patients showed lower scores, indicating an impairment compared to organized patients. In addition, for the impathy dimension of perceiving, there was a significant impairment in individuals with an unresolved attachment representation. While no difference was observed between organized controls and organized patients, there was a difference between organized and unresolved patients, suggesting that the three impathy dimensions of meta-position, accepting attitude, and understanding might be mainly affected by psychopathology, while perceiving one’s own emotions is rather a matter of attachment representation.

These findings align with previous research, which indicates that individuals with unresolved attachment representations exhibit altered processing of fear-related stimuli, particularly at the neural level (e.g., [44]). Our results complement these findings by showing that unresolved attachment is also associated with reduced behavioral recognition of fear expressions. This suggests that fear-specific impairments in unresolved attachment may manifest both neurally and behaviorally, pointing toward a broader disruption in the processing of threat-related emotional information.

In contrast, the intact recognition abilities observed in organized patients, despite the presence of psychopathology, support previous work that highlights the potential protective role of organized attachment representations in interpersonal functioning. Fonagy and Luyten [64] proposed that high levels of mentalization, typically associated with security-based attachment strategies, may promote resilience in the context of social stress and help maintain affiliative and caregiving systems, even under emotional strain.

Moreover, the observed reduction in impathy among patients aligns with previous literature linking personality disorders to diminished self-awareness and reduced capacities for self-compassion—two constructs closely related to impathy (e.g., [65,66]).

The findings of the present study indicate that an unresolved attachment exacerbates emotion-processing problems in patients with personality disorders [5,67]. Empirical support for this finding is related to neuroimaging research [68] demonstrating that unresolved attachment is uniquely associated with alterations in white matter integrity, particularly in the splenium of the corpus callosum and the inferior fronto-occipital fasciculus—structures that are crucial for emotion processing and cognitive integration. These findings suggest that unresolved attachment-related trauma may lead to structural disorganization at the neural level, potentially disrupting the processing of emotional cues and contributing to marked impairments in emotion perception.

In addition, the present study yields indicative findings that an **unresolved attachment representation is associated with a specific impaired emotion processing profile**. First, a significant impairment in facial fear recognition was observed between organized healthy subjects and unresolved attached patients. This difference was also identified as a trend between organized patients and patients with unresolved attachment representation. This finding sheds light on the specific influence of an unresolved attachment on impaired fear recognition, aligning with Bowlby’s concept of *defensive exclusion* [3]. Since, according to attachment theory, the development of a disorganized/unresolved attachment develops from a state of “*fright* without solution” [29], it is consequential that *fear* is dissociated—and thus impaired in its perception. It is interesting to recall and note here that “perceiving” was the only impathy dimension associated with an unresolved attachment. Second, the severity of unresolved attachment status (operationalized via the number of unresolved segregated systems markers in the AAP attachment narrative) was, as hypothesized, correlated with the performance of anger recognition in the unresolved patient group (but not with fear recognition performance). The more severe the unresolved attachment status, the more impaired the facial anger recognition ability. Thus, an unresolved attachment representation per se seems to be associated with impaired recognition of fear, while the severity of the unresolved attachment status was related to the anger recognition ability. The following sub-section discusses and interprets this finding in the light of attachment theory.

### 4.2. Theoretical Implications for Attachment Research

Our findings confirm the theoretical assumptions of attachment theory in relation to emotion processing. In particular, they support Bowlby’s concept of *defensive exclusion* [3]: In the case of disorganized/unresolved attachment, specific emotionally relevant information (e.g., fear or anger displayed by the attachment figure) is excluded from consciousness to make the inner experience of conflict (“fright without solution”, according to Main and Hesse [29]) bearable. In our study, this mechanism manifested itself in the observation that patients with unresolved attachment struggled to recognize the precise emotions that presumably triggered the unresolvable conflict in early childhood (fear, in the case of a frightened mother, and anger in the case of a frightening mother). This demonstrates how early childhood attachment traumas may shape the emotion processing system later on. Certain emotions are, so to speak, “blocked out” or only perceived in a biased way, which aligns with the theory of segregated affective systems. This is the first study to demonstrate the defense process of segregated systems and its connection to patterns of emotion processing in the intra- and interpersonal domains.

The result that the performance of anger recognition in unresolved patients was related to the severity level of their unresolved attachment status is an illuminating finding—as previously mentioned in the Introduction—as attachment theory [29] assumes that a disorganized/unresolved attachment develops either because the infant perceives its attachment figure as frightened (e.g., through an expression of fear resulting of a traumatized state) or frightening in the sense of being threatening (e.g., through violent behavior that is often accompanied by an expression of anger). This raises the question of whether it might be possible to identify subgroups of unresolved adult attachment along the ability continuum of anger recognition that differ in their emotion processing profile and can also be distinguished at the narrative level of the AAP attachment interview, e.g., by differentiating the markers of segregated systems (e.g., danger/failed protection, helplessness/being out of control, emptiness/isolation). The approach of AAP narrative marker differentiation has already demonstrated clinical relevance in showing distinct segregated marker profiles for different mental disorders, and also as a function of the severity of the impairment of psychological functioning [34,35,43]. However, we have to admit that a larger sample would be needed to clarify the relation between specific segregated systems’ markers and different emotion processing profiles.

Regarding the specific defensive exclusion of fear and anger, the synthesis of attachment theory and the findings of emotion research seems particularly fruitful for obtaining a deeper understanding of the emotion dynamics in unresolved attachment. Disorganized/unresolved attachment is characterized by confusion on behavioral [29] and cognitive levels [69]. In both infants and adults, disorganized/unresolved attachment shows contradictory patterns—in infants, for example, in the Strange Situation, they are seen approaching the attachment figure and simultaneously avoiding them or freezing [70]; in adults during the Adult Attachment Interview (AAI), we may observe breakdowns and incoherences in the narrative, especially with regard to topics such as loss or trauma [42]; and in the Adult Attachment Projective Picture System (AAP), we may observe emotional breakdowns like constriction during the interview. In both cases, this mirrors an inner conflict: the internalized attachment figure might be simultaneously perceived as a source of security and threat. This unsolvable dilemma results in disorganized patterns of behavior or narrative. This is where the implications of emotion science come into play: Research suggests that fear and anger represent opposing emotional forces that can result in inner conflicts in social interaction. While fear is associated with behavioral inhibition, which results in avoidance and submission, anger, on the other hand, promotes behavioral activation in the sense of confrontation and dominance [71]. Likewise, differences are shown in how these two emotions influence the individual risk preference. Anger promotes risk-seeking, and fear increases risk aversion, both at behavioral [72] and neuronal [73] levels. Thereby, it seems to be sufficient to frame anger or fear in the test subjects by presenting corresponding emotional facial expressions [74]. This shows the connection to interpersonal emotion perception and how the perception of fear and anger in others may psychologically affect ourselves.

Integrating this implication of emotion science into attachment theory helps us to understand the apparent inner conflict of unresolved attachment representation more deeply. A study by Mohammadi et al. [75] suggests that emotions segregated by attachment trauma, such as fear and anger, continue to have an unconscious effect and are linked to dissociation and complex PTSD via a disruption in emotion processing. As a result, they can generate inner conflicts that maintain or even exaggerate the symptoms. In the network model of the aforementioned study, “signs of unprocessed emotions” (e.g., unintegrated fear or anger) constitute a central pathway to dissociation symptoms and disturbances in self-organization. This suggests that segregated unresolved emotions might be considered as central nodes in the development and maintenance of trauma-related disorders.

This may explain why an increasing severity in one’s unresolved attachment status is related to an increasing impairment in one’s facial recognition of anger (which could be understood as a growing defensive exclusion of anger). As the severity of the unresolved attachment status increases, the dissociation of fear seems, therefore, to a growing extent, to be accompanied by a complementary dissociation of anger. The dissociated conflictual fear–anger interplay may exacerbate the inner conflict and, thus, intensify symptoms. That aligns with the results of a study by Gander et al. [33], showing that the severity of unresolved attachment status amplifies identity confusion in adolescents.

In this way, understanding the systemic interplay of emotions can help us to better understand the challenges people face with unresolved attachment. This argues for further interdisciplinary exchange between emotion science and attachment research, especially a deeper understanding of emotion dynamics.

### 4.3. Practical Implications for Psychotherapy of Personality Disorders

Numerous studies have shown that unified training in emotion regulation has substantial effects on reducing symptoms across psychological disorders. This has been demonstrated for depression, various forms of anxiety disorders (e.g., generalized anxiety disorder, panic disorder, social anxiety disorder), obsessive–compulsive disorder, and Borderline Personality Disorder (for a review, see [76,77,78]). Studies also demonstrated that emotion dysregulation has an inherent key function in many personality disorders beyond Borderline Personality Disorder [79]. These and other studies [80] suggest that disruptions in emotion processing could be a common denominator of various mental disorders. This notion is further supported by neuroscientific studies showing that changes in the connectivity of emotion-regulating networks in the brain are associated with a general factor of psychopathology (p-factor) [81]. But even though unified training in emotion regulation is effective, the research emphasizes the importance of a nuanced understanding of emotion processing disruptions in personality disorders [82] and, likewise, a personalization of psychotherapy for improved outcomes [83].

Furthermore, research indicates that the core of personality disorders involves difficulties in understanding and relating to oneself and others, and thus, personality disorders are often conceptualized as interpersonal disorders [84]. This notion is in line with the results of the present study, and we would add that it seems valuable for therapeutic reasons to understand them as intra-/interpersonal disorders, since our findings point out that personality disorders might impair intrapersonal emotion perception (impathy). It seems to be the additional effect of an unresolved attachment that expands the impairment to the domain of interpersonal emotion perception. Thus, the individual attachment representation may first play a key role in the sense of a protective (organized attachment) and a potential risk (unresolved attachment) factor, and second, is associated with different patterns of emotion processing in mental disorders such as personality disorders.

In our view, this leads to the following implications regarding the psychotherapy of personality disorders.

First, given the present study’s finding that an unresolved attachment representation amplifies the impairment of emotion perception, it seems be fruitful to assess the patient’s attachment representation (e.g., through the AAP attachment interview) at the beginning of a course of psychotherapy to evaluate improvement and to plan the course of the therapy in a patient-centered manner and tailor the interventions. One recent RCT study using the AAI demonstrated significant shifts from unresolved to resolved attachment in patients with Borderline Personality Disorder during Transference-Focused Psychotherapy [85]. Since the high convergence of the AAI and AAP has been demonstrated in several studies [32,86,87], the AAP seems particularly suitable for implementation in therapeutic settings [88] as it takes only about 30 min to conduct and allows us to analyze specific attachment-related trauma markers in the narratives [43,44]. This attachment assessment would help to differentiate patients with personality disorders to tailor interventions to their specific needs, especially the different impairments in emotion processing. For example, it could be relevant to focus on improving impathy in patients with organized attachment, while interpersonal emotion perception should also be addressed in patients with unresolved attachment representation. Regarding the dimensions of impathy, following the results of our study, three of them seem to be relevant to the psychotherapy of personality disorder patients with organized attachment: meta-position, accepting attitude, and understanding. There is a range of evidence-based approaches that can help to foster these impathy dimensions. For example, therapists can promote the *meta-position* ability by guiding patients to talk about their feelings in the third person or from a well-intended observer’s perspective in the therapeutic dyad. This approach is applied in Mentalization-Based Treatment (MBT) [89] and Transference-Focused Therapy (TFP) [90]. In particular, TFP treatment enables the patient to behave, mentalize, and think in a more organized and integrated fashion by working through polarized affective representations of self and significant others in the here and now of the therapeutic transference relationship. On the other hand, self-compassion interventions [91] or mindfulness-based approaches [92] foster an *accepting attitude* toward one’s own experience. By enhancing patients’ ability to engage in emotion differentiation [93], psychotherapists could improve the impathy dimension of *understanding* one’s own emotions.

Second, according to our study’s results regarding interpersonal emotion perception, fear and anger seem to be particularly affected in patients with personality disorders and unresolved attachment representation. Tailored training in recognition of these emotions in others and oneself could help to promote awareness and gradually integrate the emotional experience of these feelings through trauma-focused interventions accompanying the training, thus mitigating the attachment-related defensive processes. In this sense, in addition to the assessment of attachment representation, it would also make sense to assess the individual profile of emotion recognition (e.g., through the READ-64 test used in this study or other emotion recognition performance tests). Besides fostering the integration of segregated emotions, such tailored emotion perception training could help patients learn to use emotional and social signals better [94], improving their interpersonal interaction and understanding of social situations in everyday life. In particular, in the case of Borderline Personality Disorder, where social misunderstandings (such as misinterpreting neutral faces as angry) occur more frequently [95], this promises to attenuate conflicts and improve alliance behavior in the course of psychotherapy.

Personality disorders (PDs) impose a significant societal burden through increased healthcare utilization, reduced workplace productivity, higher rates of unemployment, and greater reliance on disability and social welfare systems. They are also associated with greater involvement in the criminal justice system and place emotional and financial strain on families and caregivers. These combined direct and indirect costs make PDs a substantial public health and economic concern. The findings of this study may offer a valuable foundation for refining therapeutic approaches for younger individuals with personality pathology, potentially contributing to improvements in broader social outcomes such as employability, reduced reliance on social welfare, and decreased involvement with the criminal justice and healthcare systems.

Beyond clinical implications, the present findings may also contribute to informing preventive efforts in educational and youth settings. Strengthening emotional self-awareness and awareness of others, as well as promoting secure attachment experiences, may represent important protective factors, particularly in light of the increasing relevance of mental health as a key resource for individual and societal functioning.

### 4.4. Limitations and Methodological Considerations

Despite the relevant theoretical and practical implications of the present study, some methodological limitations must be considered. In particular, these limitations relate to the small sample size, which might have led to insufficient power. In addition, the clinical subsample consisted of patients with personality disorders; it remains unclear to what extent the findings are transferable to other clinical groups (e.g., trauma patients without personality disorders, anxiety disorders) or mentally healthy individuals with unresolved attachment. Moreover, this study was conducted as a cross-sectional analysis, so no causal conclusions can be drawn regarding the direction of the effects. A longitudinal study could help to determine these mechanisms more precisely.

Finally, the study was partly based on self-report questionnaires (e.g., the Impathy Inventory), which may be susceptible to bias due to social desirability or impaired introspective accessibility. To reduce these limitations, more objective methods, such as psychophysiological markers or behavioral experimental designs, could be used in future studies and provide additional implications.

### 4.5. Future Research Directions

The findings of this study offer several directions for future research, particularly with regard to refining our understanding of differential patterns in emotion processing as they relate to attachment representation. While this study focused on segregated systems as a defense mechanism in unresolved attachment representation, future research should extend these considerations to the other defense processes described by Bowlby [3] and further operationalized by George and West [32] in the AAP—namely, *Deactivation* and *Cognitive Disconnection*. Examining whether these defense processes are also associated with distinct patterns of emotion recognition could provide a more nuanced perspective of the relationship between attachment and emotion processing.

Beyond further analysis of attachment-related emotion processing, another promising line of research involves the nonverbal expressions of emotion during attachment-related narratives. The present study relied on behavioral performance in emotion recognition as the primary outcome measure; however, given the link between emotion and facial expressions [96], examining how facial expressions unfold while participants engage in attachment narratives within the AAP would be particularly relevant. Specifically, future studies should investigate whether individuals with unresolved attachment exhibiting impaired recognition of fear (and associated with the severity of unresolved attachment status impaired recognition of anger) also show differences in spontaneous facial expressions of emotions when narrating attachment-relevant themes. This could provide further insight into whether the defensive exclusion of specific emotions (e.g., fear or anger) is reflected in reduced or inhibited facial expressions of these emotions.

A particularly compelling approach would be to distinguish microexpressions (facial expressions that occur faster than 500 ms) from macroexpressions (facial expressions that occur longer than 500 ms) during the AAP, as microexpressions have been shown to indicate emotions that are either outside of conscious awareness or are deliberately suppressed [54]. Given that unresolved attachment is theorized to involve dissociated affective states (cf. segregated systems), it would be valuable to explore whether individuals who defensively exclude fear or anger also exhibit microexpressions (instead of macroexpressions) of these emotions. This line of inquiry could bridge the gap between attachment research and the field of emotion science by integrating both verbal (narrative content) and nonverbal (facial expressions) indicators of emotion processing. In this context, recent advances in artificial intelligence and computer vision may offer valuable tools for the automated analysis of nonverbal behavior. For example, AI-based systems could be used to detect microexpressions or quantify emotion-specific facial muscle activity during attachment narratives, thereby increasing the objectivity and scalability of assessing nonverbal indicators of emotion processing in future studies.

A combination of neuroimaging, psychophysiological markers, and behavioral data could provide a comprehensive understanding of how attachment-related defensive processes shape emotion recognition, impathy, and emotion processing in general. By integrating these perspectives, future research can deepen our theoretical understanding of attachment-related emotion processing while simultaneously enhancing the precision of diagnostic assessments and interventions in psychotherapy.

## 5. Conclusions

In summary, this study demonstrates that adult attachment representations are critical determinants of both self- and other-focused emotion processing in the context of personality disorders.

Specifically, patients with organized attachment representations showed preserved interpersonal emotion recognition but significantly impaired impathy, indicating that organized attachment may offer a protective buffer for recognizing others’ emotions while self-focused emotion perception remains affected by psychopathology. In contrast, unresolved attachment was associated with impairments in both domains, particularly with strongly reduced fear recognition. Furthermore, the severity of unresolved attachment status correlated with poorer anger recognition, suggesting that dissociation of specific emotions may follow distinct defensive exclusion patterns depending on attachment-related trauma severity. These findings underscore the importance of assessing and addressing attachment-related emotion processing patterns, as well as explicitly identifying the individual‘s attachment representation, as part of the diagnostic process. This enables psychodynamic or attachment-informed psychotherapy, in which emotion-focused interventions can be more precisely adapted to the patient’s internal working models of attachment and related disruptions in emotion processing. Such targeted personalization may increase therapeutic effectiveness, particularly in individuals with unresolved attachment and severe impairments in emotional functioning.

## Figures and Tables

**Table 1:** Sociodemographic and clinical characteristics of the patient, control, and total groups.

Characteristic	Patients	Controls	Total
**Age (years)**, *M* (*SD*)	21.33 (2.35)	22.00 (1.32)	21.52 (2.12)
**Gender**, *n* (%)			
Female	15 (62.5)	8 (88.9)	23 (69.7)
Male	8 (33.3)	1 (11.1)	9 (27.3)
Non-binary	1 (4.2)	–	1 (3.0)
**Marital Status**, *n* (%)			
Single	19 (79.2)	6 (66.7)	25 (75.8)
Married	2 (8.3)	–	2 (6.0)
Long-term relationship	3 (12.5)	3 (33.3)	6 (18.2)
**Level of education**, *n* (%)			
No compulsory schooling	1 (4.2)	–	1 (3.0)
Compulsory schooling	12 (50.0)	–	12 (36.4)
Apprenticeship	2 (8.3)	–	2 (6.0)
Technical/commercial school	1 (4.2)	–	1 (3.0)
University entrance qualification	6 (25.0)	6 (66.7)	12 (36.4)
Bachelor’s degree	2 (8.3)	3 (33.3)	5 (15.2)
**Country of origin**, *n* (%)			
Austria	19 (79.2)	–	19 (57.6)
Germany	2 (8.3)	8 (88.9)	10 (30.4)
Other	3 (12.5)	1 (11.1)	4 (12.0)
**Attachment Representation**, *n* (%)			
Organized	11 (45.8)	9 (100.0)	20 (60.6)
Unresolved	13 (54.2)	–	13 (39.4)
Unresolved Trauma Markers, *M (SD)*	3.25 (4.45)	0 (0)	2.36 (4.05)
**SCL-90-S (GSI)**, *M* (*SD*)	1.71 (0.61)	0.35 (0.09)	1.34 (0.80)

Notes. *n* = Number of participants; % = percentage of participants.

**Table 2:** Unadjusted mean values and standard deviations (SDs) of the different attachment groups, including statistics from the general linear models used to test for group differences, for the Impathy Inventory, READ-64, and SCL-90-S.

Measure/Dimension	Organized Controls *n* = 9	Organized Patients *n* = 11	Unresolved Patients *n* = 13 ^1^	General Linear Model
**Impathy Inventory**, *M* (*SD*)	**61.44 (8.28) ^a^**	**39.73 (16.16) ^b^**	**28.45 (14.74) ^b^**	*F*_1, 28_ = 14.39, *p* < 0.001, *η*^2^*_p_* = 0.51 *
Perceiving	15.78 (2.64) ^a^	13.00 (4.22) ^a^	9.45 (4.30) ^b^	*F*_1, 28_ = 6.74, *p* = 0.004, *η*^2^*_p_* = 0.33 *
Meta-Position	14.78 (3.31) ^a^	8.18 (3.40) ^b^	5.82 (3.87) ^b^	*F*_1, 28_ = 16.55, *p* < 0.001, *η*^2^*_p_* = 0.54 *
Accepting Attitude	15.22 (1.92) ^a^	8.18 (5.72) ^b^	4.82 (4.49) ^b^	*F*_1, 28_ = 13.73, *p* < 0.001, *η*^2^*_p_* = 0.50 *
Understanding	15.67 (2.65) ^a^	10.36 (4.54) ^b^	8.36 (3.67) ^b^	*F*_1, 28_ = 9.72, *p* < 0.001, *η*^2^*_p_* = 0.41 *
**READ-64**, *M* (*SD*)	**59.72 (9.17) ^a^**	**56.54 (12.06) ^a^**	**47.36 (8.71) ^b^**	*F*_1, 30_ = 4.63, *p* = 0.02, *η*^2^*_p_* = 0.24 *
Fear	52.78 (28.49) ^a^	39.77 (20.78) ^a,b^	22.12 (19.20) ^b^	*F*_1, 30_ = 5.11, *p* = 0.01, *η*^2^*_p_* = 0.25 *
Surprise	82.10 (18.92) ^a^	85.23 (23.60) ^a^	84.62 (18.51) ^a^	*F*_1, 30_ = 0.06, *p* = 0.94, *η*^2^*_p_* = 0.00
Anger	66.67 (13.98) ^a^	55.68 (25.84) ^a^	47.12 (25.59) ^a^	*F*_1, 30_ = 1.90, *p* = 0.17, *η*^2^*_p_* = 0.11
Disgust	43.06 (30.69) ^a^	59.09 (30.15) ^a^	40.93 (23.80) ^a^	*F*_1, 30_ = 1.42, *p* = 0.26, *η*^2^*_p_* = 0.09
Contempt	44.44 (27.32) ^a^	44.32 (29.24) ^a^	33.65 (25.20) ^a^	*F*_1, 30_ = 0.61, *p* = 0.55, *η*^2^*_p_* = 0.04
Sadness	65.28 (19.54) ^a^	57.60 (23.23) ^a^	43.30 (30.45) ^a^	*F*_1, 30_ = 2.15, *p* = 0.13, *η*^2^*_p_* = 0.13
Happiness	56.35 (25.00) ^a^	39.77 (31.53) ^a^	44.23 (27.77) ^a^	*F*_1, 30_ = 0.89, *p* = 0.42, *η*^2^*_p_* = 0.06
Social Smile	66.67 (25.00) ^a^	70.46 (27.54) ^a^	63.25 (25.09) ^a^	*F*_1, 30_ = 0.23, *p* = 0.80, *η*^2^*_p_* = 0.02
**SCL-90-S (GSI)**, *M* (*SD*)	**0.35 (0.09) ^a^**	**1.52 (0.58) ^b^**	**1.86 (0.61) ^b^**	*F*_1, 30_ = 24.17, *p* < 0.001, *η*^2^*_p_* = 0.62 *

Notes. GSI = Global Severity Index; * *p*-value < 0.05; ^1^ The data for the impathy inventory were missing for two subjects. ^a,b^ Groups with values that do not share a superscript within the same line of text are significantly different from each other. Bold values indicate the total scores (Impathy Inventory, READ-64) or the Global Severity Index (SCL-90-S), presented to enhance readability.

## Data Availability

The data presented in this study are available on request from the corresponding author due to the ethical considerations of the Medical University of Innsbruck.

## Appendix A. Group Comparisons on the Four Impathy Dimensions

To complement the main analyses, Figure A1 presents the mean scores of the three attachment-related groups (organized controls, organized patients, and unresolved patients) across the four dimensions of the Impathy Inventory. The subscales include *perception, meta-position*, *accepting attitude*, and *understanding*. The graphs illustrate group differences as identified in the pairwise comparisons.
